# Ten simple rules for optimal and careful use of generative AI in science

**DOI:** 10.1371/journal.pcbi.1013588

**Published:** 2025-10-28

**Authors:** Mohamed Helmy, Lingling Jin, Amr Alhossary, Tamer Mansour, Diogo Pellagrina, Kumar Selvarajoo

**Affiliations:** 1 Vaccine and Infectious Diseases Organization (VIDO), University of Saskatchewan, Saskatchewan, Canada; 2 Vaccinology and Immunotherapeutics Program, School of Public Health, University of Saskatchewan, Saskatchewan, Canada; 3 Department of Computer Science, University of Saskatchewan, Saskatchewan, Canada; 4 Department of Computer Science, Idaho State University, Boise, Idaho, United States of America; 5 Bioinformatics Institute (BII), Agency for Science, Technology and Research (A*STAR), Singapore, Republic of Singapore; 6 Chemistry Department, Wesleyan University, Middletown, Connecticut, United States of America; 7 Department of Population Health and Reproduction, University of California, Davis, California, United States of America; 8 Department of Clinical Pathology, College of Medicine, Mansoura University, Mansoura, Egypt; 9 Synthetic Biology Translational Research Program, Yong Loo Lin School of Medicine, National University of Singapore (NUS), Singapore, Republic of Singapore; 10 Synthetic Biology for Clinical and Technological Innovation (SynCTI), National University of Singapore (NUS), Centre for Life Sciences #02-07, Singapore, Republic of Singapore; 11 School of Biological Sciences, Nanyang Technological University (NTU), Singapore, Republic of Singapore; Dassault Systemes BIOVIA, UNITED STATES OF AMERICA

## Introduction

Modern AI technologies leverage natural language processing (NLP), a subfield of AI dedicated to understanding, interpreting, and generating human language for developing large language models (LLMs), which have significantly advanced the capabilities of AI systems [1]. These models can perform complex language tasks such as text generation, summarization, translation, and sentiment analysis, with unprecedented accuracy. The two main kinds of pre-training LLMs are the BERT-like models (e.g., BioBERT [2], proteinBERT [3], and PubMedBERT [4]) used primarily for language understanding; and the GPT-like models (e.g., BioGPT [5] and ChatGPT-4o) used primarily for language generation [5].

Towards the end of 2022, a new wave of highly efficient generative AI (GenAI) tools caused a paradigm shift in content generation. The new tools, powered by LLMs, became available on platforms, such as OpenAI’s ChatGPT and Google’s Gemini, and have been integrated into tools for the automation of text generation, writing assistance, content summarization, and data analysis workflow development. For instance, GPT-3.5 and later versions have demonstrated the ability to generate text for various applications, ranging from drafting essays to answering technical questions with a level of relevance to the context that was never achieved with previous models [6]. Similarly, other platforms leverage AI to enhance written communication by providing real-time grammar, style, clarity suggestions, and content generation based on a prompt [7]. Importantly, while these platforms are grounded in generative models, many of them now integrate or enable advanced data processing and analytics capabilities such as machine learning (ML) techniques, and embedding-based fine-tuning which extend their functionality beyond text or image generation. This broader scope reflects how GenAI is used in practice within research workflows, and it is in this sense that we discuss its applications throughout the rules.

These advances in GenAI extend rapidly into scientific research and biomedical applications. For instance, SciSpace Copilot assists researchers in interpreting scientific literature by providing plain-language explanations of figures and methods (https://scispace.com). Similarly, Ought’s Elicit employs GenAI to support literature reviews, offering automated extraction of key findings, claims, and summaries from published research (https://elicit.com). DeepMind’s AlphaFold has significantly contributed to the structural biology field by accurately predicting the 3D structures of over 200 million proteins, accelerating protein research and drug discovery workflows [8]. In the biomedical field, BioMedLM is a domain-specific language model designed to assist with tasks like biological question answering and literature summarization [9]. New applications are also emerging in data visualization, where GenAI tools are helping researchers generate intuitive representations of complex datasets, and in research integrity, where AI detectors are being explored as tools to identify potential misuse of GenAI in scientific writing [10]. Overall, these tools illustrate how GenAI is being embedded in the scientific workflow, enhancing research productivity, knowledge discovery, and accessibility.

## Concerns about using GenAI in scientific research

**Fig 1:**
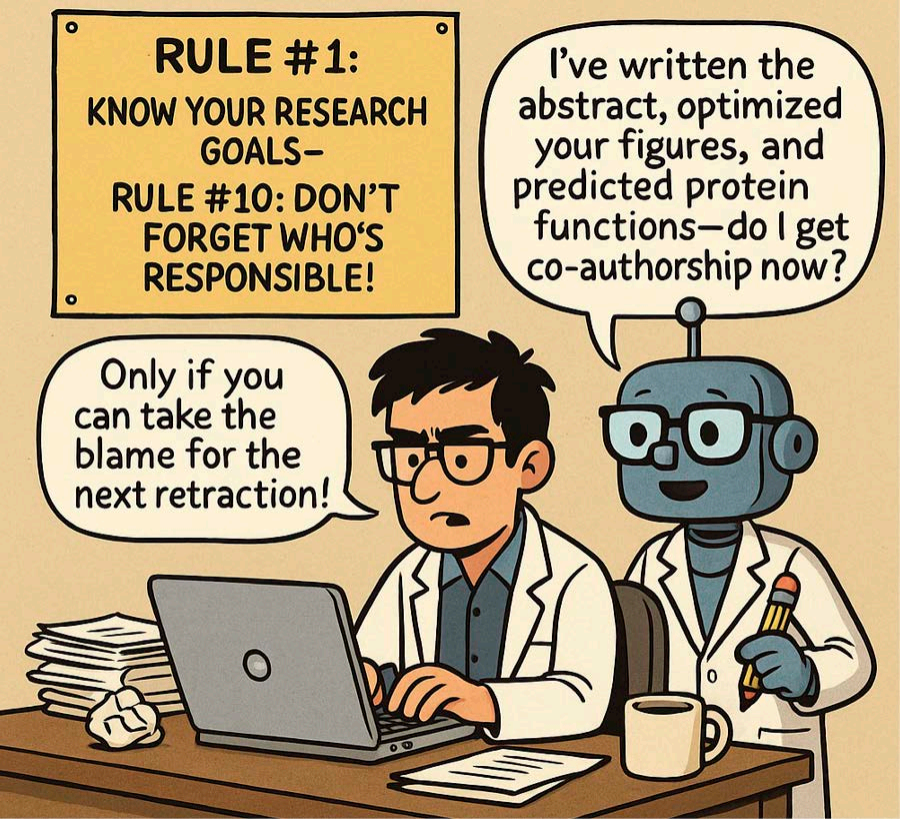
Even the smartest AI can’t sign the ethics form. The figure was generated using ChatGPT.

The rise of GenAI use in scientific research has brought transformative opportunities, but it also raises several valid concerns that must be addressed to ensure ethical and responsible use. While GenAI has the potential to accelerate scientific discovery, its rapid adoption, without accompanying frameworks, guidelines, or adequate user training, introduces challenges that could undermine research integrity, ethical standards, and public trust. Some of the key concerns associated with the use of GenAI in research and its implications on scientific community and society at large are:

1. *Mass Generation of Low-Quality Content:* GenAI tools have intensified the problem of paper mills by enabling the rapid creation of fake or low-quality research, including fabricated data, images, and entire manuscripts [11,12]. This has led to a growing concern over declining publication standards [13].
2. *AI as an Author*: The appearance of ChatGPT as an author on early articles raised serious ethical concerns [14,15], as AI cannot meet authorship criteria such as accountability or consent. While some initial preprints listed ChatGPT as a co-author, this practice is now widely rejected by journals and publishers as inconsistent with established authorship standards [16] (Fig 1).
3. *Risk of Plagiarism:* AI-generated text, if used without proper revision or disclosure, may include unoriginal or copyrighted content, raising serious plagiarism concerns [17]. GenAI can also be misused to fabricate results or bypass plagiarism detection by rephrasing existing text in ways that evade traditional checking tools [18,19].
4. *AI Hallucinations* (inaccuracy of the generated contents): GenAI tools can produce convincing but entirely fabricated information, a phenomenon known as AI hallucination [20]. This is especially evident in fake references or citations [21], undermining the reliability of AI-generated scientific content. While newer tools have improved factual accuracy, hallucinations remain persistent issues [22].
5. *Bias Amplification*: GenAI tools are based on LLMs trained on vast amounts of data. The training datasets dominate the quality and accuracy of the generated content. Therefore, if the training dataset is unintendedly biased, it is expected that the AI systems can amplify the existing biases, leading to skewed research outcomes, particularly in socially sensitive domains [23]. The training datasets could be unintendedly biased when they have one or more of the following problems: a) lack of diversity or representation, which introduces cultural biases related to gender, age, race, or religion, b) ambiguous labelling, where the data labelling process is subjective resulting in biases based on characteristics like race or gender, or c) reinforcing stereotypes, where the training data contains stereotypes bias related to culture or gender [24]. The existing biases in the training datasets result in bias amplification or the introduction of new bias(es), which undermines the GenAI outcomes. For instance, a review of AI-based tools in global health highlighted how racial and geographic biases in training data can severely limit the generalizability and equity of medical AI solutions [25].
6. *Unethical Use of GenAI*: Using GenAI in university admissions, assignments, or scholarship applications undermines merit-based evaluations and is widely considered unethical [26,27]. Such misuse has prompted many educational institutions to implement strict guidelines, treating unauthorized AI use as a form of plagiarism to preserve fairness and academic integrity (S1 Table).
7. *Security and Safety Concerns*: Many GenAI platforms reserve the right to use user data for model training, raising serious concerns about privacy, data security, and unauthorized access [28–30]. This is especially risky for researchers or organizations handling sensitive or unpublished content, as third-party access could lead to data breaches or misuse of proprietary information [28].
8. *Intellectual Property (IP) and Originality Concerns*: Uploading original content such as unpublished data or research proposals to GenAI platforms risks that content being incorporated into future outputs, leading to potential IP disputes and loss of originality [31]. To protect confidentiality and ownership, institutions like NIH and Canada’s Tri-Agency have issued strict policies prohibiting the use of GenAI with sensitive or proprietary research materials [32].
9. *Long-Term Implications on Research Skills*: Growing reliance on GenAI in research risks diminishing essential skills like critical thinking, hypothesis generation, coding, and scientific writing [33,34]. As AI handles more cognitive tasks, researchers may lose opportunities to develop foundational competencies, potentially widening the gap between AI-savvy scientists and those who depend on its applications.

Addressing these concerns is vital to safeguarding the integrity and credibility of scientific research while harnessing the benefits of GenAI.

## Responsible use of GenAI in scientific research

As the integration of GenAI tools into scientific research raises concerns that threaten research integrity, various organizations have taken steps to establish regulations and guidelines to ensure their responsible use. Governments, universities, funding agencies, working groups, school boards, and publishers have all recognized the need for ethical standards to balance the potential benefits of GenAI with the challenges it presents (Tables 1–3 and S1). These efforts aim to address issues such as transparency, accountability, and data security, which are critical for maintaining the integrity of research.

**Table 1:** Selected policies and guidelines by governments and funding agencies to regulate the use of GenAI tools in research.

Type	Institution/Organization	Region	Remarks
Governmental	Government of Canada	Canada	Advisory only, FASTER Principles
	The White House	USA	TAKEN DOWN by new administration by the time of submission to remove barriers
	Department of Education	USA	A comprehensive governance framework that ensures responsible AI adoption while aligning with federal mandates and fostering collaboration
	European Commission	EU	Tied to the EU AI Act, establishing guidelines for the responsible use of GenAI in research within the European Research Area with integrity and human oversight
	Council of Higher Education	Turkey	Emphasizes balancing technological advancements with scientific integrity, accountability, and ethical responsibility
	Australian Government - OAIC	Australia	Emphasizes compliance with Australia’s Privacy Act 1988 when using commercial AI products, ensuring privacy-by-design
	MEXT	Japan	Has a strong focus on AI literacy and ethical AI use in education, emphasizing the responsible integration of AI in schools while safeguarding students’ rights and privacy
Funding Agencies	CIHR-College of Reviewers	Canada	Preventing the use of GenAI tools in the research proposals review process
	Tri-Agency	Canada	Provide guidance on the responsible use of GenAI in the development and review of research grant proposals
	NIH	USA	Explicitly prohibiting AI tools from being used to analyze or generate review content to protect confidentiality and integrity
	European Research Council	EU	AI tools must not replace humans in ERC grant evaluations, ensuring accountability for proposals and review processes

MEXT: Ministry of Education, Culture, Sports, Science and Technology, OAIC: Office of the Australian Information Commissioner.

**Table 2:** Selected policies and guidelines by educational institutions to regulate the use of GenAI tools in research.

Type	Institution/Organization	Region	Remarks
Educational	Peel District School Board	Canada	It covers key areas such as ethical AI usage, instructional applications, assessment practices, content development, privacy, security, and accessibility
	New York City Public Schools	USA	Prevented the use of GenAI in all school-related activities, then allowed it with restrictions
University	University of Saskatchewan	Canada	A comprehensive, cautious, and ethics-driven guide, emphasizing critical evaluation, academic integrity, and responsible AI use, balancing innovation with potential risks
	Harvard University	USA	It stands out for building on existing university regulations while emphasizing responsible use, data privacy, security, compliance, and academic integrity, requiring clear AI usage expectations in academics, restricting confidential data input, and warning against AI-related cybersecurity threats
	Singapore Management University	Singapore	Analyzing how researchers disclose AI use in academic manuscripts, highlighting best practices, trends, and the importance of transparency in maintaining research integrity
	MIT	USA	Focuses on balancing innovation with responsibility, providing flexible guidelines that encourage the ethical use of GenAI while maintaining academic integrity and human oversight
	Melbourne Institute of Technology	Australia	Provides detailed guidance on the ethical use of GenAI tools in academic work, emphasizing the importance of understanding both their capabilities and limitations
	University of Toronto	Canada	Discipline-specific AI guidelines, recognizing the diverse impacts of GenAI across academic fields, promoting ethical use, transparency & academic integrity
	University of Adelaide	Australia	Explicitly guiding students on responsible AI use while integrating it into research and learning with strong emphasis on academic integrity
	Ignacio Chávez National Institute of Cardiology	Mexico	Emphasizes the ethical and regulatory challenges of AI in cardiology, advocating for transparency, clinical validation, and the responsible integration of AI in medical research and practice
	Oxford University	UK	Focuses on ensuring responsible AI use in communications, emphasizing accuracy, transparency, and human oversight to maintain trust and credibility in public messaging
	Imperial College London	UK	Had strong emphasis on developing AI literacy, guiding students and staff on how to critically assess, responsibly use, and transparently acknowledge GenAI at work
	The University of Edinburgh	UK	Providing practical guidance on the ethical and responsible use of Generative AI while emphasizing the importance of understanding their limitations and advising students to critically assess AI-generated content

**Table 3:** Selected policies and guidelines by publishers and editorial associations to regulate the use of GenAI tools in research.

Type	Institution/Organization	Region	Remarks
Publisher	CAAA Journals	Canada	It requires authors to disclose the use of generative AI in the creation of manuscripts, including detailing its role in the methods section, while adhering to guidance from the Committee on Publication Ethics (COPE)
	JAMA Network	USA	Explicitly prohibiting AI tools from being authors, requiring transparency in AI use for writing and research, and mandating human accountability for all content
	Nature	UK	It emphasizes the importance of transparency and integrity in scientific research by prohibiting the attribution of authorship to AI tools like ChatGPT and requiring researchers to document any use of AI in the methods or acknowledgments sections of their publications
	Elsevier	The Netherlands	It requires the disclosure of the use of AI tools in their manuscripts, promoting transparency and accountability in research, and ensuring compliance with the tools’ terms of use
	IEEE Publications	USA	It mandates that any use of AI-generated content in submissions must be fully disclosed in the acknowledgements section, with specific citation of the AI system used
	Frontiers	Switzerland	It stands out for requiring full transparency in AI use, prohibiting AI tools as co-authors, mandating disclosure of AI-generated content, ensuring human oversight in peer review, and encouraging authors to share AI prompts and outputs for greater accountability
	Cell Press	USA	Presents a clear stance that AI tools cannot be authors, strictly requires transparency in AI-assisted content creation, and it emphasizes human responsibility for accuracy
	SciOpen	China	It emphasizes responsible AI usage by disallowing AI as an author, requiring disclosure of AI tool usage in manuscript preparation, and aligning with COPE publishing guidelines
	The Lancet	USA	Explicitly prohibiting the use of GenAI for creating or altering medical images, figures, and patient-related content while mandating full transparency in AI-assisted writing
	European Respiratory Society	EU	Carefully defines the responsible and ethical use of GenAI for writing support, requiring authors to acknowledge any AI involvement, while emphasizing the importance of authorship responsibility and adherence to privacy and scientific integrity standards
	Public Library of Science (PLoS)	USA	Introduced a policy that requires authors to disclose any use of AI tools in their research or article content and ensure the accuracy of information and proper attribution of any ideas generated by AI tools. The policy further outlines the actions to be taken if concerns arise regarding the use of GenAI tools
Other*	International Committee of Medical Journal Editors	USA	It requires authors to disclose AI usage in manuscript preparation, while also clarifying that AI cannot be an author due to its lack of responsibility for content accuracy and integrity
	Committee on Publication Ethics	UK	It emphasizes that AI cannot be credited as authors in academic publications since it lacks legal and ethical responsibilities, and authors must disclose how AI tools were used in their work, ensuring transparency while remaining fully accountable for the content produced
	World Association of Medical Editors	Worldwide	It states that only humans can be authors of scholarly works and mandates transparency about the use of AI tools, while emphasizing the responsibility of authors to ensure the accuracy and integrity of content generated or influenced by AI, alongside the need for editors and peer reviewers to disclose any use of AI in manuscript evaluation
	European Association of Science Editors	EU	Provides detailed, discipline-agnostic recommendations on AI use in scholarly communication, emphasizing transparency, accountability, and ethical considerations while aligning with global publishing standards

* Others indicate an organization that is outside the five categories of institutions listed in the table, such as an NGO or an Editors' association.

For example, the Government of Canada has published a guide for AI use across governmental institutions [35]. The guidelines introduce the FASTER principle, which stands for Fair, Accountable, Secure, Transparent, Educated, and Relevant. The guidelines serve as a foundation for developing institution-specific policies and practices, promoting ethical GenAI usage across sectors. According to the guidelines, users are advised to critically assess whether AI is necessary to meet organizational or user needs, recognizing its role as a complementary tool rather than a substitute for human decision-making. These principles complement well-established data governance frameworks such as FAIR (Findable, Accessible, Interoperable, and Reproducible) [36]. FAIR guides how we manage and share data, while FASTER shapes how we apply AI technologies to those data and to scientific processes. Similar to the Government of Canada, the US government [37], the European Commission [38], and many other governments published GenAI usage policies and guidelines (Tables 1 and S1).

Several universities and research institutions also present policies and guidelines for regulating the use of GenAI tools in the research and education processes (Tables 2 and S1). One of the most comprehensive guides is the University of Saskatchewan Library Guide on Generative Artificial Intelligence [39]. The guide provides a detailed introduction to the GenAI technology, independent guidelines and instructions for educators, students, researchers and administration, and detailed guides on utilizing GenAI for studying, teaching, searching, researching, writing, publishing and creating where the questions of “Should I use GenAI for each of these activities” is answered in details with a graphical flowchart that helps the user find the answer that supports responsible use in GenAI [39]. S1 Table lists over 40 different guidelines and policies of universities and research institutions worldwide.

Similarly, major research funding and regulation agencies like the National Institutes of Health (NIH) [40] in the United States, the Tri-Agency [32] in Canada and the European Research Council (ERC) in the European Union [41] have implemented rules that limit or prevent the use of AI in specific contexts, such as the review of grant proposals, to prevent potential biases and conflicts of interest and protect IP. For instance, CIHR College of Reviewers completely prevents the use of AI tools in the revision process of the research grant proposals and warns against the use of AI tools during the grant proposal preparation [42].

Various academic journal publishers have also responded to the increasing use of GenAI tools in research and academic writing by requiring authors to disclose the use of AI tools in the preparation of manuscripts (Tables 3 and S1). Some journals even mandate that authors specify how GenAI tools were employed, ensuring accountability and upholding ethical writing practices [43–47].

These proactive measures by key stakeholders in scientific research and publication highlight the importance of fostering a responsible GenAI ecosystem in research by adhering to established guidelines and participating in the ongoing dialogue about AI ethics to harness the power of GenAI while protecting the values of scientific inquiry and integrity.

## Ten simple rules for the responsible use of generative AI

**Fig 2:**
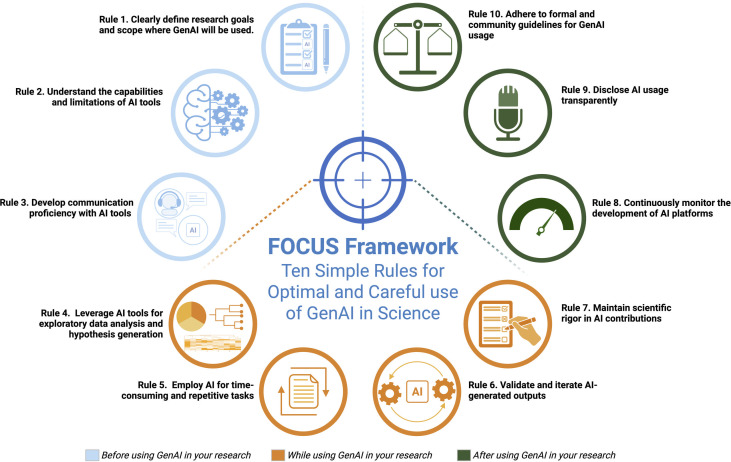
The FOCUS Framework for Optimal and Careful Use of GenAI in Science. The FOCUS framework has 10 steps that represent a structured approach to integrating GenAI into scientific research responsibly and ethically. The steps are categorized into three colour-coded categories indicating when each step is applied (before, while, or after the use of GenAI in your research). The figure was created using Biorender.com.

Based on the review of over 100 policies and guidelines (S1 Table), we propose the FOCUS Framework (Framework for Optimal and Careful Use of AI in Science) to efficiently harness the power of the new GenAI technologies in scientific research and academic writing while maintaining ethical use and research integrity (Fig 2). The FOCUS Framework consists of ten simple rules that make it compatible with the policies and guidelines set by different institutions, which help the researchers leverage GenAI technologies responsibly while upholding the standards of ethical and scientific rigor.

To develop the FOCUS Framework and its ten guiding rules, we began by analyzing a curated sample of GenAI policies and guidelines from leading institutions across several sectors, including research funders, universities, academic journals, and government agencies. This initial sample was selected to represent major institutions with broad influence in their respective domains. From this analysis, we drafted a preliminary set of ten rules that reflected common ethical concerns, best practices, and shared values. We then expanded our review to include over 100 policies and guidelines from diverse global sources, using this broader dataset to assess whether any major positions contradicted or added nuance to our initial rules. This second-stage analysis allowed us to iteratively refine and adapt the framework, ensuring that it accommodated special requirements and avoid conflict with edge cases, not fully captured in the initial sample. The final framework thus reflects both foundational consensus and alignment with the current landscape of GenAI governance in research and education. AI tools such as ChatGPT and DeepSeek were used in these analyses. A list of reviewed guidelines and institutions is provided in S1 Table.

### Before using GenAI in your research

#### Rule 1. Clearly define research goals and scope where GenAI will be used.

Before integrating GenAI into your research, it is essential to clearly outline the specific goals and scope of the study. This involves identifying the research questions, objectives, and stages of the research process where GenAI will be applied, such as data analysis, hypothesis generation, or manuscript preparation. By explicitly defining the role of GenAI, researchers can ensure that its use aligns with the study’s overall design and avoids unnecessary or inappropriate applications. This clarity fosters a structured approach to leveraging AI’s strengths while maintaining focus on the core scientific objectives.

Avoiding unnecessary applications of GenAI is not merely about efficiency but also about safeguarding research integrity and responsible resource use. Deploying GenAI in situations where it adds little scientific value can introduce ethical risks, such as biased or inaccurate outputs, while also consuming computational resources and potentially contributing to environmental costs. Moreover, overreliance on GenAI for routine tasks that researchers can and should perform independently may erode essential skills in critical thinking, data analysis, and scientific writing. Therefore, “unnecessary applications” are those in which GenAI neither enhances scientific outcomes nor justifies the associated risks and costs.

#### Rule 2. Understand the capabilities and limitations of AI tools.

To effectively utilize GenAI in research, it is crucial to understand its capabilities and limitations within the specific context of your study. AI tools excel at tasks like processing large datasets, generating text, and identifying patterns, but they may also produce biased, incomplete, or inaccurate outputs due to the limitations of their training data or algorithms. Researchers must critically assess whether the selected AI tool is suitable for their objectives and remain cautious about over-reliance. Recognizing these strengths and constraints helps mitigate risks of misuse or misinterpretation.

#### Rule 3. Develop communication proficiency with AI.

Effective communication with GenAI tools is essential to maximize their utility in research. Researchers must develop skills to craft clear, specific, and unbiased prompts that guide the AI to produce relevant and high-quality outputs. This involves understanding how to frame questions, provide contextual information, and refine iterative queries to achieve the desired results. Additionally, researchers should learn to interpret and critically evaluate AI responses, using them as a complement to their expertise rather than a substitute. Mastery of AI communication fosters efficient collaboration with these tools, enhancing their contribution to research processes such as hypothesis generation, data analysis, and reporting.

### While using GenAI in your research

#### Rule 4. Leverage AI tools for exploratory data analysis and hypothesis generation.

GenAI tools can be very useful for exploratory data analysis and the early stages of hypothesis development due to its capabilities to identify patterns, trends, or anomalies in large datasets. Thus, these tools help researchers uncover insights that might otherwise remain hidden. For instance, AI can cluster data points, visualize relationships, or generate potential research questions based on observed patterns. However, researchers must critically assess AI-generated hypotheses and ensure that they are grounded in empirical evidence or theoretical frameworks before pursuing them (see below). Leveraging AI in this way enables a more efficient and informed approach to designing experiments and advancing scientific inquiry.

#### Rule 5. Employ AI for time-consuming and repetitive tasks.

GenAI tools are well-suited for automating repetitive and time-consuming tasks, freeing researchers to focus on more complex and creative aspects of their work. These tools can streamline processes like data cleaning, error correction, and formatting standardization, ensuring datasets are ready for robust analysis. They can also assist in annotating large datasets, such as labelling biological sequences, categorizing images, or tagging textual data, which accelerates workflows significantly. Beyond data preprocessing, AI can also automate routine administrative or documentation tasks, enhancing efficiency across research operations. Again, it is essential to validate all AI-generated outputs rigorously to maintain accuracy and reliability, as errors in foundational tasks such as data cleaning can undermine subsequent research findings.

#### Rule 6. Validate and iterate AI-generated outputs.

Ensuring the reliability and accuracy of AI-generated outputs is a critical step in integrating GenAI into research workflows. Researchers should adopt an iterative process, refining prompts and reviewing multiple outputs to enhance the quality and relevance of the AI’s contents. All AI-generated insights, whether they involve data analysis, hypothesis generation, or visualizations, should be cross-verified against empirical evidence, domain expertise, or established scientific knowledge. Iterative validation helps to identify potential inaccuracies, biases, or inconsistencies in the outputs, reducing the risk of misinterpretation or flawed conclusions. This approach fosters trust in the use of AI tools within scientific practices.

#### Rule 7. Maintain scientific rigor in AI contributions.

To maintain research integrity, scientific rigor must remain a cornerstone when incorporating AI-generated content into research. In addition to critically evaluating all AI contributions, including text, data analyses, and visualizations, AI outputs should be treated as supplementary tools rather than definitive conclusions, requiring thorough review and, where necessary, corroboration with independent methods. The research questions, main findings and study conclusions should all be done through the researchers, not the AI tools.

### After using GenAI in your research

#### Rule 8. Continuously monitor the development of AI platforms.

Integrating AI into research workflows requires ongoing assessment of its performance, accuracy, and relevance to the study objectives. With the rapid development of GenAI platforms such as the introduction of new services and the improvements of existing ones, researchers should routinely update their knowledge on the platforms/tools they are using and the new ones as well. This includes evaluating the AI tools they use and its updates to identify potential biases, inaccuracies, or ethical concerns that could come with the new services and could compromise their research integrity. Regular updates to AI models or algorithms should be scrutinized to understand their impact on outputs. In addition, follow the development and improvements of the AI detectors and once they are reliable enough, integrate them in your workflow. Overall, this helps researchers ensure that these tools remain effective to their research and aligned with the evolving demands of scientific inquiry.

#### Rule 9. Disclose AI usage transparently.

Transparency in the use of GenAI tools is essential to uphold the integrity and reproducibility of scientific research. Researchers should explicitly disclose where and how AI tools have contributed to their work, such as in data preprocessing, hypothesis generation, analysis, or manuscript preparation. This includes specifying the AI platform, version, and the nature of its involvement. Clear disclosure not only ensures ethical compliance but also allows peers to assess the reliability and reproducibility of the research. Furthermore, it is getting more and more common for journals to ask for disclosure or a GenAI usage statement (S1 Table).

#### Rule 10. Adhere to formal and community guidelines for GenAI usage.

Researchers must align their use of GenAI with established ethical standards and community-specific guidelines. This includes adhering to institutional policies, funding agency requirements, and journal standards regarding AI applications in research. Ethical considerations should prioritize data privacy, intellectual property rights, and avoiding harm or bias in AI-generated outputs. By following these guidelines, researchers contribute to responsible use of GenAI in research that maintains integrity, transparency, and fairness in the scientific community.

The FOCUS framework emphasizes defining research goals, understanding AI limitations, critically reviewing AI-generated content, ensuring transparency in AI contributions, and adhering to institutional and ethical guidelines. By following this structured approach, researchers can harness the power of AI while maintaining the rigor, originality, and ethical integrity of their work. As governments, universities, funding agencies, and publishers establish policies to regulate AI in scientific research, continued collaboration and dialogue will be essential to align AI adoption with best practices. The responsible integration of AI must prioritize human oversight, continuous evaluation, and adherence to ethical principles to prevent misuse. Moving forward, research institutions are recommended to foster AI literacy and develop policies that support responsible AI usage while minimizing risks to scientific credibility. By balancing innovation with accountability, the research community can ensure that AI serves as a valuable tool for accelerating discovery without compromising the fundamental principles of science.

## Supporting information

S1 Table Over 100 policies and guidelines published by governments, universities, funding agencies, and publishers worldwide to regulate the use of GenAI tools in research and education. (XLSX)
